# Liver Injury in COVID-19 Infection: A Systematic Review

**DOI:** 10.7759/cureus.9487

**Published:** 2020-07-31

**Authors:** Atit Ghoda, Manoj Ghoda

**Affiliations:** 1 General Internal Medicine, East Surrey Hospital/Surrey and Sussex Healthcare National Health Service Trust, Redhill, GBR; 2 Gastroenterology and Hepatology, Gujarat Superspeciality Clinic, Ahmedabad, IND

**Keywords:** covid-19, elevated liver associated enzymes, lft - liver function tests

## Abstract

The severe acute respiratory syndrome coronavirus 2 (SARS-CoV-2) or novel coronavirus disease (COVID-19) pandemic is sweeping through the world. The overwhelming pathology seems to be in the upper and lower respiratory tract; however, the involvement of other organs, including the liver, has also been reported.

Whether liver enzyme derangement is a common feature of COVID-19 is not known. For those patients who have concomitant liver enzyme derangement with COVID-19, the prevalence, extent, and rate of progression to liver failure is not known. In view of unclear evidence regarding this, we conducted a systematic review of the literature on liver injury in COVID-19 patients.

The aim of this review was to ascertain whether liver enzyme derangement is a common feature in adult patients with confirmed COVID-19 infection, determine the relation of deranged liver enzymes with outcome or mortality in COVID-19, and determine if liver failure is a common feature of COVID-19.

The PubMed and OVID Medline databases were searched systematically. Cross-sectional studies and case-control studies involving adult patients with confirmed COVID-19 and having data on liver enzymes were included. Independent extraction of the data was done by two independent authors.

A total of 23 articles were identified by the initial filtering search. Abstracts were reviewed and screened to shortlist studies. A full-text assessment of the shortlisted articles for eligibility criteria identified five articles. Manual searching via the LitCovid (National Library of Medicine tool) search hub produced a further two studies that were eligible.

Many of the COVID-19 patients in the various studies had a varying degree of deranged liver enzymes. The degree of injury was mild in most cases; and it appears to correlate with the severity of COVID-19 infection. Severe liver injury causing significant liver damage, liver failure, or death is uncommon.

The main limitations of the study were the heterogeneity of studies and incomplete data on the trajectory of liver tests during the disease course as well as the final outcomes of the patients in the studies.

## Introduction and background

A pandemic caused by the severe acute respiratory syndrome coronavirus 2 (SARS-CoV-2) or novel coronavirus disease (COVID-19) started in December 2019 in the Wuhan province of China and swept through the world by April 2020, affecting 187 of the 192 countries of the world with varying severity [1-4].

The most common clinical presentation leading to hospital admission is respiratory tract involvement with fever, shortness of breath, and cough associated with radiographical findings consistent with pneumonia [5].

The spectrum of COVID-19 presentation is wide, with very mild cases at one end having minor symptoms like a sore throat and loss of smell or taste [6], to illness resulting in death at the other end. Apart from respiratory tract involvement, other organ injuries, including acute kidney injury, liver damage, cerebrovascular stroke, and gastroenteritis are also reported [5,7]. The involvement of the liver in adult patients with COVID-19 infection has been reported, however, the exact prevalence of liver abnormalities, the type of liver enzyme derangement, and its relationship with outcomes is not clear yet.

We aimed to systemically review the existing literature, to determine the extent of liver enzyme derangement in COVID-19 and its relationship with the severity of disease and overall prognosis. We particularly focused on the following tests: alanine aminotransferase (ALT) (reference range: 0-35 units/L), alkaline phosphatase (ALP) (reference range: 36-92 units/L), and total bilirubin (reference range: 5.1-20.5 µmol/L). Where available, we also included aspartate aminotransferase (AST) (reference range: 0-35 units/L) and gamma-glutamyltransferase (GGT) (reference range 0-30 units/L).

The following are the specific questions this review attempts to answer. In symptomatic adults with COVID-19 infection, is liver enzyme derangement a common feature? In those with deranged liver enzymes and COVID-19, how common is progression to liver failure? Is there a relationship between liver enzyme derangement and disease outcome?

## Review

We conducted a search using the Preferred Reporting Items for Systematic Reviews and Meta-Analyses (PRISMA) methodology. Two authors (AMG and MKG) performed a systematic search on the following data platforms: PubMed and OVID Medline. Studies were identified using an electronic search database. The search was applied to the OVID Medline (2019 - present) database and PubMed (2019 - present).

The search details column for PubMed included covid, covid 19, coronavirus, liver, liver injury, liver function, liver function tests/ing, liver enzyme/s, acute liver failure, liver failure. The complete search strategy, as well as the procedure used in the systematic review, has been reported in the Appendix.

Prospective and retrospective cross-sectional studies and case-control studies involving adult patients with COVID-19, which included data on liver enzymes/liver function tests, met the inclusion criteria. The searches were restricted to publications in English. The following studies were excluded: case reports, editorials, commentaries, opinions, reviews, meta-analyses, and systematic reviews, overlapping or duplicate studies, studies involving only pregnant women and children, controlled and uncontrolled drug or intervention trials, duplicate studies, and studies involving specific subgroups only.

Abstracts of the studies were screened independently by AMG and MKG to decide on eligibility. In case of a dispute about the eligibility of a study, this was resolved by AMG and MKG discussing the merits/demerits of inclusion/exclusion.

Search results

A total of 23 articles were identified by the initial filtering search. A review and screening of abstracts were done to shortlist studies. A full-text assessment of the shortlisted articles for eligibility criteria identified five articles. Manual searching via the LitCovid (National Library of Medicine tool) search hub produced a further two studies that were eligible.

We developed a data extraction sheet based on the Cochrane Consumers and Communication Review Group’s data extraction template. The data extraction sheet was initially piloted for three studies and modifications were made accordingly.

The following information was extracted: country of study, study setting [hospital, community, intensive care unit (ICU)], study design, sample size, the definition of abnormal liver tests, the prevalence of abnormal liver tests, relationship with the outcome, and reports of acute liver failure

The following are key points from the literature review:

A cross-sectional study by Cai Q et al. looked at the prevalence of liver test abnormalities in 417 COVID-19 positive patients at a single center in China [8]. The median age of the study population was 47 (34-60) years. Patients were classified as having mild or severe COVID-19 based on the clinical exam, symptoms, and chest radiography findings. Seventy-eight point two percent (78.2%) patients had mild disease, and 21.8% of patients had severe COVID-19 disease. Liver test abnormality was defined as elevation in the following parameters: ALT >40 U/L, AST >40 U/L, gamma-glutamyl transferase (GGT) >49 U/L, alkaline phosphatase (ALP) >135 U/L, and total bilirubin (TBIL) >17.1 μmol/L. Liver injury was defined as ALT and/or AST over 3× upper limit of normal (ULN), ALP, GGT, and/or TBIL over 2× ULN. On presentation to the hospital, 41% of patients had abnormal liver tests and 5% had a liver injury. During the entire period of hospitalization, 76.3% of patients developed abnormal liver tests, and 21.5% developed a liver injury. Ten patients suffered from multiorgan failure and three patients died. Two patients of these 10 were reported to have had liver failure, however, this seems to be in the context of multiorgan failure rather than isolated organ dysfunction.

Fan Z et al. conducted a retrospective single-center study of 148 patients with confirmed COVID-19 in Shanghai, China [9]. The mean age of the population was 50 years. Abnormal liver function was defined as any parameter (ALT, AST, ALP, GGT, and total bilirubin) more than the upper limit of the normal lab reference value. Fifty-five (37.2%) had abnormal liver function at hospital admission. The percentage of patients developing abnormal liver function tests after admission was not reported, nor was the type and severity of the liver enzyme derangement. Patients with abnormal liver function had longer mean hospital stays (15.09 ± 4.79 days) than patients with normal liver function (12.76 ± 4.14 days) (P =.021). One death was reported in the study. No cases of acute liver failure were reported.

A multicenter study of 70 COVID-19 patients over nine centers in China was conducted by Qi et al. [10]. Sixty-seven (95.71%) patients had non-severe COVID-19 disease, and three (4.29%) had severe disease on admission. One death was reported during the course of the study. Liver injury was defined as having elevated ALT, AST, and total bilirubin. Thirty-two (45.71%) patients with COVID-19 were classified as having a liver injury on admission. No cases of acute liver failure were reported. At the time of publication in March, the authors reported no significant difference in the length of hospital stay amongst patients with and without liver injury.

Singh et al. conducted a multi-center research network study with regards to clinical characteristics and outcomes of COVID-19 among patients with pre-existing liver disease in the United States [11]. The authors performed a real-time search and analysis of electronic medical records of 37 health organizations using specialized software. They identified 2780 patients with COVID-19 (age >10 years) and stratified them into two groups based on the presence (liver disease (LD), n=250 patients) or absence (non-LD, n=2530) of liver disease. ALT elevations (>50 U/L) were seen in 46.1% in the LD group and 50.6% in the non-LD group. The type and extent of liver enzyme derangement were not described. There was no mention of any instances of acute liver failure.

Xie et al. report a retrospective study of 79 COVID patients in a non-ICU ward in China [12]. The median age was 60 years. Thirty-one point six (31.6%) patients had elevated ALT, 35.4% of patients had elevated AST, and 5.1% had elevated bilirubin. Patients with abnormal liver tests had a longer length of stay in hospital (15.4 days (11.0‐16.8)) as compared to the group without abnormal liver tests (11.4 days (8.5‐14.0)). There were no deaths in the entire cohort. There were no reported instances of acute liver failure.

Zhang et al. conducted a case-control type study of 240 patients [13]. This was done at a single center in China, with the patients notably consisting only of infected staff and their families. One-hundred-fifteen adult patients were COVID-19 positive. 119 patients admitted with community-acquired pneumonia were reviewed as the control group. On admission, elevated ALT was observed in 9.57% of COVID-19 patients. Only one patient presented a >3 × ULN elevation of ALT. In comparison with CAP patients, there was no significant difference in the levels of ALT. Elevated total bilirubin was observed in 6.96% of COVID‐19 patients as compared to 6.14% in community-acquired pneumonia (CAP) patients. Five-point twenty-one (5.21%) of COVID‐19 patients had mild ALP elevation as compared to 15.79% in the CAP patients. The mean ALT level in severe COVID-19 patients was higher than in mild disease (37.87 ± 32.17 vs. 21.22 ± 12.67), and the mean total bilirubin level was higher in severe COVID-19 disease than in mild disease (14.12 ± 6.37 vs 10.27 ± 4.26, P < .001). Seven patients in the COVID-19 group became critically unwell and one patient died. There was no significant change in ALT, total bilirubin, or ALP in these patients post-admission to ICU. There were no reports of acute liver failure. Histopathological exam of the liver of the patient who died showed mild sinusoidal dilatation and minimal lymphocytic infiltration only.

 Phipps et al. performed a multicentre retrospective cohort study of 2273 COVID-19 patients from three centers in the USA [14]. The median age was 65. Liver injury was defined and categorized as mild (<2 times ULN), moderate (2-5 times ULN), and severe (>5 times ULN). Five percent of the cohort had chronic liver disease. Of all COVID-positive patients, 45% had mild, 21% moderate, and 6.4% had a severe liver injury. Moderate and severe liver injury was more common in patients who required ICU-level care. Multivariate analysis showed that severe acute liver injury was associated with elevated inflammation markers, including ferritin and interleukin 6 (IL-6). The proportion of patients who were very unwell seems to be higher in this study, seeing that 23% of the patients required ICU-level care. Amongst patients with severe liver injury (n=145), 69% required ICU care.

Discussion

The prevalence of deranged liver enzymes in patients with COVID-19 showed a wide variation in the studies included. It can be readily seen that in most patients, the liver enzyme derangement seems to be mild in nature. Although some of the studies indicate that patients with more severe COVID-19 disease may have a higher prevalence of liver enzyme derangement, acute liver failure seems to be very rare. 

Multiple theories have been proposed to explain the mechanism of liver injury.

The SARS-CoV-2 virus has been shown to use the angiotensin-2 converting enzyme (ACE2) as a means for cell entry. In healthy liver tissue, ACE2 is expressed mainly on cholangiocytes [15]. This form of direct viral entry could possibly lead to the liver enzyme derangement seen in COVID-19 patients.

In addition, immune-mediated inflammation, such as a cytokine storm, might be a critical factor associated with disease severity and mortality [16]. Immune dysfunction, including lymphopenia or immune system overreaction, which accompanies disease progression, can also independently lead to liver derangement.

Pneumonia-associated hypoxia and hypotension might also contribute to liver injury [17] or even develop into liver failure in patients with COVID-19 who are critically ill.

Lastly, many of these patients are critically ill and in ICU and maybe on a variety of medications or even parenteral nutrition. It is, therefore, also possible that the liver impairment is partly, if not wholly, due to these additional factors, which might explain the large variation observed across the different cohorts.

There is a need for further research to delineate the exact mechanisms by which this disease causes liver enzyme impairment.

Clinical implications

Physicians should be aware that patients with COVID-19 may have deranged liver enzymes associated with their illness. This should be kept in mind, especially if the patient is commenced on medication or therapies that might have a potential for hepatotoxicity. One should also look for features that might suggest pre-existing or undiagnosed liver disease in these patients. Acute liver failure seems to be rare and is described only in isolated case reports [18].

It is important to note that co-morbidities of those at high risk for severe COVID-19 also overlap with non-alcoholic fatty liver disease (NAFLD) and a few other advanced liver diseases, so they must be identified proactively, and hypoxia and hypotension, which may impair liver further, maybe energetically treated.

## Conclusions

Deranged liver enzymes are not an uncommon finding in COVID-19 patients. Usually, it is in the form of altered aminotransferases picked up during routine investigations. Seldom does it present with acute hepatitis. The cause of the liver injury is not clearly established, but most likely, it seems multifactorial, with a cytokine storm and immune dysregulation possibly playing a role so it could be hypoxia, hypotension, multiple drugs, direct viral effect, and ICU-related infections. In the majority of patients, the liver injury seems to be self-limiting, not requiring any specific intervention, and not associated with acute liver failure.

## Figures and Tables

**Figure 1:**
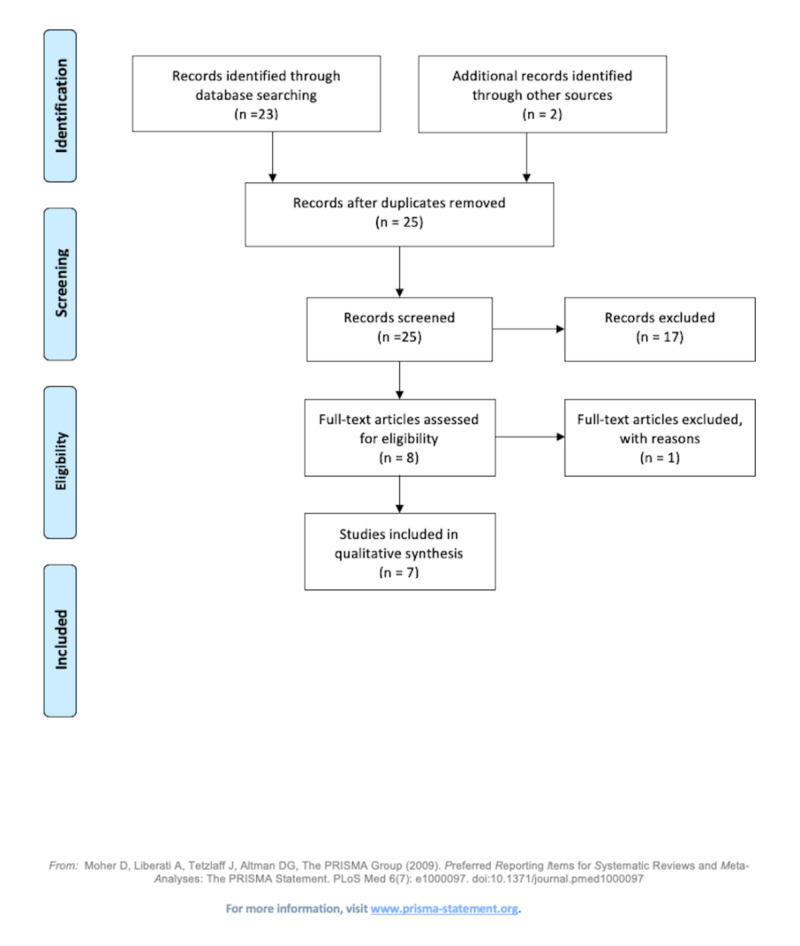
Flow chart for the systematic review Flow chart for the procedure used in the systematic review [19]

## Appendices

Complete search strategy for the PubMed search

 ((((((liver[Title] OR liver injury[ti]) OR liver function[Title]) OR (liver function test[ti] OR liver function testing[ti] OR liver function tests[ti])) OR (liver enzyme[ti] OR liver enzymes[ti])) OR acute liver failure[ti]) OR liver failure[ti]) AND ((covid[Title] OR covid 19[Title]) OR coronavirus[Title])

Procedure used in the systematic review

## References

[1] (2020). WHO Director-General's opening remarks at the media briefing on COVID-19. https://www.who.int/dg/speeches/detail/who-director-general-s-opening-remarks-at-the-media-briefing-on-covid-19---11-march-2020.

[2] Velavan TP, Meyer CG (2020). The COVID-19 epidemic. Trop Med Int Health.

[3] World Health O (2020). WHO. Coronavirus disease 2019 (COVID-19): situation report, 73. https://apps.who.int/iris/handle/10665/331686.

[4] Organization WH. (1 (2020). WHO. Q&A on coronaviruses (COVID-19). http://www.emro.who.int/health-topics/corona-virus/questions-and-answers.html.

[5] Guan W-J, Ni Z-Y, Hu Y (2020). Clinical characteristics of coronavirus disease 2019 in China. N Engl J Med.

[6] Patel A, Charani E, Ariyanayagam D, Abdulaal A, Denny SJ, Mughal N, Moore LSP (2020). New-onset anosmia and ageusia in adult patients diagnosed with SARS-CoV-2 infection. Clin Microbiol Infect.

[7] Huang C, Wang Y, Li X (2020). Clinical features of patients infected with 2019 novel coronavirus in Wuhan, China. Lancet.

[8] Cai Q, Huang D, Yu H (2020). COVID-19: abnormal liver function tests. J Hepatol.

[9] Fan Z, Chen L, Li J (2020). Clinical features of COVID-19-related liver damage. Clin Gastroenterol Hepatol.

[10] Qi X, Liu C, Jiang Z (2020). Multicenter analysis of clinical characteristics and outcome of COVID-19 patients with liver injury. J Hepatol.

[11] Singh S, Khan A (2020). Clinical characteristics and outcomes of COVID-19 among patients with pre-existing liver disease in United States: a multi-center research network study. Gastroenterology.

[12] Xie H, Zhao J, Lian N, Lin S, Xie Q, Zhuo H (2020). Clinical characteristics of non-ICU hospitalized patients with coronavirus disease 2019 and liver injury: a retrospective study. Liver Int.

[13] Zhang Y, Zheng L, Liu L, Zhao M, Xiao J, Zhao Q (2020). Liver impairment in COVID-19 patients: a retrospective analysis of 115 cases from a single centre in Wuhan city, China. Liver Int.

[14] Phipps MM, Barraza LH, LaSota ED (2020). Acute liver injury in COVID‐19: Prevalence and association with clinical outcomes in a large US cohort. Hepatology.

[15] Chai X, Hu L, Zhang Y (2020). Specific ACE2 expression in cholangiocytes may cause liver damage after 2019-nCoV infection [PREPRINT]. bioRxiv.

[16] Alqahtani SA, Schattenberg JM (2020). Liver injury in COVID- 19: the current evidence. United European Gastroenterol J.

[17] Chand N, Sanyal AJ (2007). Sepsis-induced cholestasis. Hepatology.

[18] Weber S, Mayerle J, Irlbeck M, Gerbes AL (2020). Severe liver failure during SARS-CoV-2 infection. Gut.

[19] Moher D, Liberati A, Tetzlaff J, Altman DG, PRISMA Group (2009). Preferred reporting items for systematic reviews and meta-analyses: the PRISMA statement. PLoS Med.

